# Cytokinome Profile of Patients with Type 2 Diabetes and/or Chronic Hepatitis C Infection

**DOI:** 10.1371/journal.pone.0039486

**Published:** 2012-06-20

**Authors:** Susan Costantini, Francesca Capone, Eliana Guerriero, Raffaele Marfella, Angela Sorice, Patrizia Maio, Michele Di Stasio, Giuseppe Paolisso, Giuseppe Castello, Giovanni Colonna

**Affiliations:** 1 INT “G. Pascale" - Oncology Research Centre of Mercogliano, Mercogliano, Italy; 2 Department of Geriatrics and Metabolic Diseases, Second University of Naples, Naples, Italy; 3 Unità Operativa Malattie Infettive, Azienda Ospedaliera di Rilievo Nazionale “San Giuseppe Moscati", Avellino, Italy; 4 Institute of Food Science - CNR, Avellino, Italy; 5 Department of Biochemistry and Biophysics and Interdepartmental Research Center for Computational and Biotechnological Sciences, Second University of Naples, Naples, Italy; University of Medicine and Dentistry of New Jersey - New Jersey Medical School, United States of America

## Abstract

Both type 2 diabetes (T2D) and chronic hepatitis C (CHC) infection are associated with increased risk of developing hepatocellular carcinoma (HCC). Cytokines are known to play an important role not only in the mechanisms of insulin resistance and glucose disposal defects but also in the pathological processes occurring in the liver during viral infection. We evaluated the serum levels of many cytokines, chemokines, adipokines and growth factors in patients with type 2 diabetes, CHC, CHC-related cirrhosis, CHC and type 2 diabetes and CHC-related cirrhosis and type 2 diabetes by BioPlex assay. The obtained data evidenced that the serum levels of some proteins are significantly up-regulated in all the patients or in those with only one disease and are often higher, even if in different amounts, when both diseases are associated. In particular, our results can be useful for the clinical monitoring of patients because they give specific information in regard to the progression from CHC to LC and CHD to LCD. Moreover, some molecules have shown significant correlations with clinical/biochemical data, suggesting the possibility to define mini-panels that can be used as specific markers for the different disease staging. However, our observations demonstrate that an integrated approach is much more powerful than isolated measurements to evaluate specific stages of these two complex pathologies (type 2 diabetes and chronic CHC hepatitis) alone or when they are concomitant in a patient. In fact it has emerged as an accurate, simple, specific, noninvasive, reproducible and less expensive method that, in future, could be included in routine clinical practice to monitor the association of type 2 diabetes and/or CHC to liver cirrhosis and, possibly, to cancer, and to improve the prognosis of these diseases.

## Introduction

Recent epidemiological studies suggest that profound alterations in glucose homeostasis, as occurs in type 2 diabetes mellitus or metabolic syndrome, increase the risk of fatty liver disease as well as of hepatocellular carcinoma (HCC) [1]. In fact, many clinical and experimental data have demonstrated that type 2 diabetes is more common among patients with chronic hepatitis C virus than in those with hepatitis B virus or in the general population [2]–[4] and that CHC infection is associated with increased risk of developing insulin resistance and its major late feature, type 2 diabetes [5]–[6].

In general, CHC infection itself also increases the risk of HCC, leading to complications such as chronic hepatitis, hepatocellular necrosis and inflammation, fibrosis, cirrhosis and HCC. For patients with CHC-related cirrhosis the risk for development of HCC is 0.54 to 2.0% per year [7].

Recently type 2 diabetes has been recognized as a cofactor that can modify the course of CHC infection and can be used as an independent predictor of HCC. Recent studies have shown that type 2 diabetes was associated with hepatocarcinogenesis in patients with CHC infection without liver cirrhosis [8]–[9]. Therefore, there has been a great interest in the search of possible specific immunological markers able to follow the progression of CHC to cirrhosis and HCC also in association with type 2 diabetes.

It is well known that cytokines and chemokines are signaling molecules involved in the inflammatory as well as in cancer-related processes [10]. Hence, understanding in patients affected from cancers the dynamics of the complex interaction network of cytokines [11], defined “cytokinome" [12], should be very useful to follow the disease progression and evolution from its early stages as well as to define therapeutic strategies by using systems biology approaches [11]. However, many data on cytokines have been reported in the literature and sometimes their correlation appears to be difficult. In fact, in the most part of these papers, the researchers have evaluated only few cytokines by single measurements and, therefore, both the global inflammation mechanism and the cytokine relationships are understandable with difficulty [12]–[13].

In our approach, the serum levels of a panel of numerous cytokines, chemokines, adipokines and growth factors were evaluated at the same time by BioPlex assay in patients with chronic hepatitis C (CHC), CHC-related cirrhosis (LC), type 2 diabetes (T2D), CHC hepatitis and type 2 diabetes (CHD), CHC-related cirrhosis and type 2 diabetes (LCD) and in healthy controls, to identify those molecules that might be useful for discriminating the various stages of these diseases. In other hands, the simultaneous quantitative determination of a large panel of cytokines, able to report the correct ratios and dynamics between highly and poorly represented molecules, has emerged as an accurate, simple, specific, noninvasive, reproducible and less expensive method that, in future, could be included in routine clinical practice to monitor the association of type 2 diabetes and/or CHC to liver cirrhosis and, possibly, to cancer, and to improve the prognosis of these diseases.

## Materials and Methods

### Patients

In this study we enrolled seventeen patients (6 women, 11 men) with type 2 diabetes (T2D), twenty patients (11 women, 9 men) with CHC, twenty patients (12 women, 8 men) with LC, ten patients (4 women, 6 men) with CHD, ten patients (4 women, 6 men) with LCD, and 20 healthy control subjects (11 women, 9 men). In Table 1 we report the clinical characteristics of all patients. This choice depended from our interest to study the association of chronic liver damage leading to cirrhosis in presence or absence of type 2 diabetes. The ADA criteria were used to classify patients with the type 2 diabetes [14]: i) fasting plasma glucose 126 mg/dl (7.0 mmol/l) where fasting is defined as no caloric intake for at least 8 h or ii) symptoms of hyperglycemia and a casual plasma glucose 200 mg/dl (11.1 mmol/l) where casual is defined as any time of day without regard to time since last meal whereas the classic symptoms of hyperglycemia include polyuria, polydipsia, and unexplained weight loss, or iii) 2-h plasma glucose 200 mg/dl (11.1 mmol/l) during an OGTT where the test has been performed as described by the World Health Organization, using a glucose load containing the equivalent of 75 g anhydrous glucose dissolved in water.

**Table 1:** Biochemical characteristics of all patients.

	T2D	CHC	CHD	LC	LCD	Control range
**Age**	61,8	62,54	65,2	70	71,8	60,92
**Gender**	11M/6F	10M-10F	7M-3F	8M-12F	5M-5F	9M-11F
**Hypertension**	17	10	3	11	3	
**Hypercholesterolemia**	17	5	6	5	7	
**Cigarette smoking**	12	n.d.	n.d.	n.d.	n.d.	
**BMI (kg/m^2^)**	27,4±1	24,2±0,9	28,1±1,2	24,4±0,8	27,9±1,4	<25
**Total cholesterol (mg/dL)**	192±21	170±5,8	181±4,6	182±7,2	200±2,1	130–200
**Triglycerides (mg/dL)**	179±21	98±23	111±35	105±27	117±36	40–165
**AST (IU/L)**	31±4	71,43±2,3	66,4±4,5	81,25	112,1	5–40
**ALT (IU/L)**	33±6	121,1	104	71,96	106,7	7–56
**TotBilirubin (mg/dL)**	1,01±0,06	0,94	1,03	1,72	1,58	0,20–1,30
**Albumin (g/dL)**	4,2±0,9	4,01	4,44	2,61	3,4	3,5–5
**PLT (mL)**	198464±10221	187413	162000	113875	114833	150000–400000
**CHC – PCR RNA**	negative	positive	positive	positive	positive	
**CHC genot**	negative	1∶11; 2∶9	1∶6; 2∶4	1∶12; 2∶8	1∶7; 2∶3	
**Glycemia (mg/dL)**	145,9±12,3	86	139,2	88,3	132,5	70–105
**Creatinine (mg/dL)**	0,9±0,01	0,9	0,8	1	1,1	0,6–1,36
**AFP (ng/mL)**	<10	<10	<10	<20	<20	
**Child Pügh**				A:8; B:7;C:5	A:3; B3;C:4	

§ n.d.  =  not detected.

We report the number of patients to which some parameters refer. For clinical data the mean value and the related control range, evaluated in healthy donors, are shown.

Moreover, the cirrhosis was defined by clinical diagnosis in presence of liver functional insufficiency (Child Pügh  =  B and C) or by liver biopsies in the case of initial chronic hepatopathy without clear liver laboratoristic function tests of cirrhotic evolution (Child Pügh = A). All patients with CHC, LC, CHD and LCD had transaminase values (AST and ALT) higher than control range, evaluated in healthy donors. Moreover, liver cirrhosis patients presented higher birilubin values and lower albumin and platelets count (PLT) respect to control range. Both CHD and LCD had hyperglycemia. All patients had normal alpha-fetoprotein (AFP) values and did not present cancer. The patients with T2D, CHD and LCD were overweight with BMI values in the range between 25–29 kg/m^2^ whereas those with only CHC and LC have BMI of about 25 kg/m^2^. The smoking status was known only for T2D patients.

For this study we obtained ethics approval from the ethics committee at our institution (INT “G. Pascale" – CROM) and obtained written informed consent from all involved participants.

### BioPlex Assay

Blood samples were collected from a peripheral vein and kept on ice. Serum was collected by centrifugation (3,000 rpm for 10 min at 4°C), aliquoted, and stored at −80°C until analyzed. A multiplex biometric ELISA-based immunoassay, containing dyed microspheres conjugated with a monoclonal antibody specific for a target protein was used according to the manufacturer’s instructions (Bioplex, Bio-Rad Lab., Inc., Hercules, CA, USA). Soluble molecules were measured using three commercially available kits: i) 21-Plex panel: IL-1α, IL-2R, IL-3, IL-12p40, IL-16, IL-18, CCL27, CXCL1, CXCL9, CXCL12, HGF, IFN-α2, LIF, MCP-3, M-CSF, MIF, β-NGF, SCF, SCGF-β, TNF-β, TRAIL; ii) 10-Plex panel: C-peptide, ghrelin, GIP, glp-1, glucagon, insulin, leptin, PAI-1, resistin, visfatin and iii) 2-Plex panel: adiponectin and adipsin.

Each experiment was performed in duplicate using the same procedure described in our recent papers [10], [13]. Serum levels of all proteins were determined using a Bio-Plex array reader (Luminex, Austin, TX) that quantifies multiplex immunoassays in a 96-well plate with very small fluid volumes. The analytes concentration was calculated using a standard curve, with software provided by the manufacturer (Bio-Plex Manager Software).

### Data Analysis and Statistics

The nonparametric Mann-Whitney U test was used to evaluate differences between cytokine, chemokine and growth factor ratios in the patients and healthy controls. In particular p<0.05 is indicated with *, p<0.01 with **, and p<0.001 with ***. T-test was used to compare the serum levels of these proteins evaluated in different patients groups. The correlations between the cytokine levels and clinical/biochemical data were determined using the Pearson correlation coefficient. Values of p<0.05 were considered to be statistically significant. Multivariate logistic regression analysis was performed to evaluate associations between levels of significant cytokines/adipokines and smoking status, hypertension, BMI, and hypercholesterolemia. Odds ratios (OR) and 95% confidence intervals (95% CI) for association between each cytokine and four parameters were estimated using unconditional logistic regression models. In details, OR represent a measure of the strength of association between variables and, when OR is greater then 1, it indicates that the association is significant. The statistical programs Prism 4 (GraphPad Software, San Diego, CA, USA) and MedCalc (MedCalc software, Mariakerke, Belgium) were employed.

## Results

### Comparison between Patients with Type 2 Diabetes and Healthy Donors

**Figure 1:**
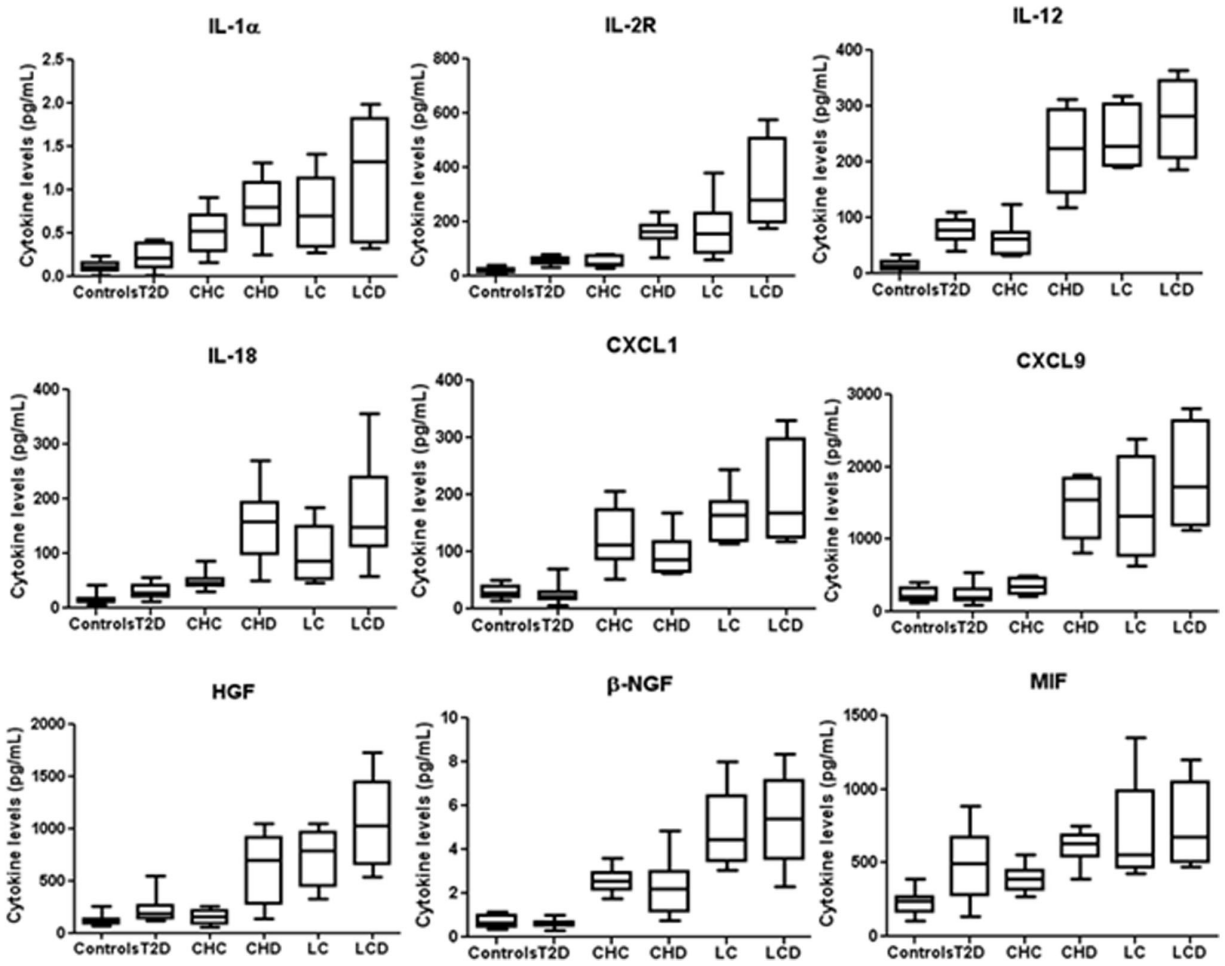
Significant cytokines in some patient groups. We report the significant molecule levels from controls and patients with type 2 diabetes (T2D), chronic hepatitis C (CHC), CHC-related cirrhosis (LC), CHC hepatitis and type 2 diabetes (CHD) and CHC-related cirrhosis and type 2 diabetes (LCD), evaluated by 21-Plex panel, are plotted with box-and-whisker graphs. The boxes extend from the 25th to the 75th percentile, and the line in the middle is the median. The error bars extend down to the lowest value and up to the highest.

**Figure 2:**
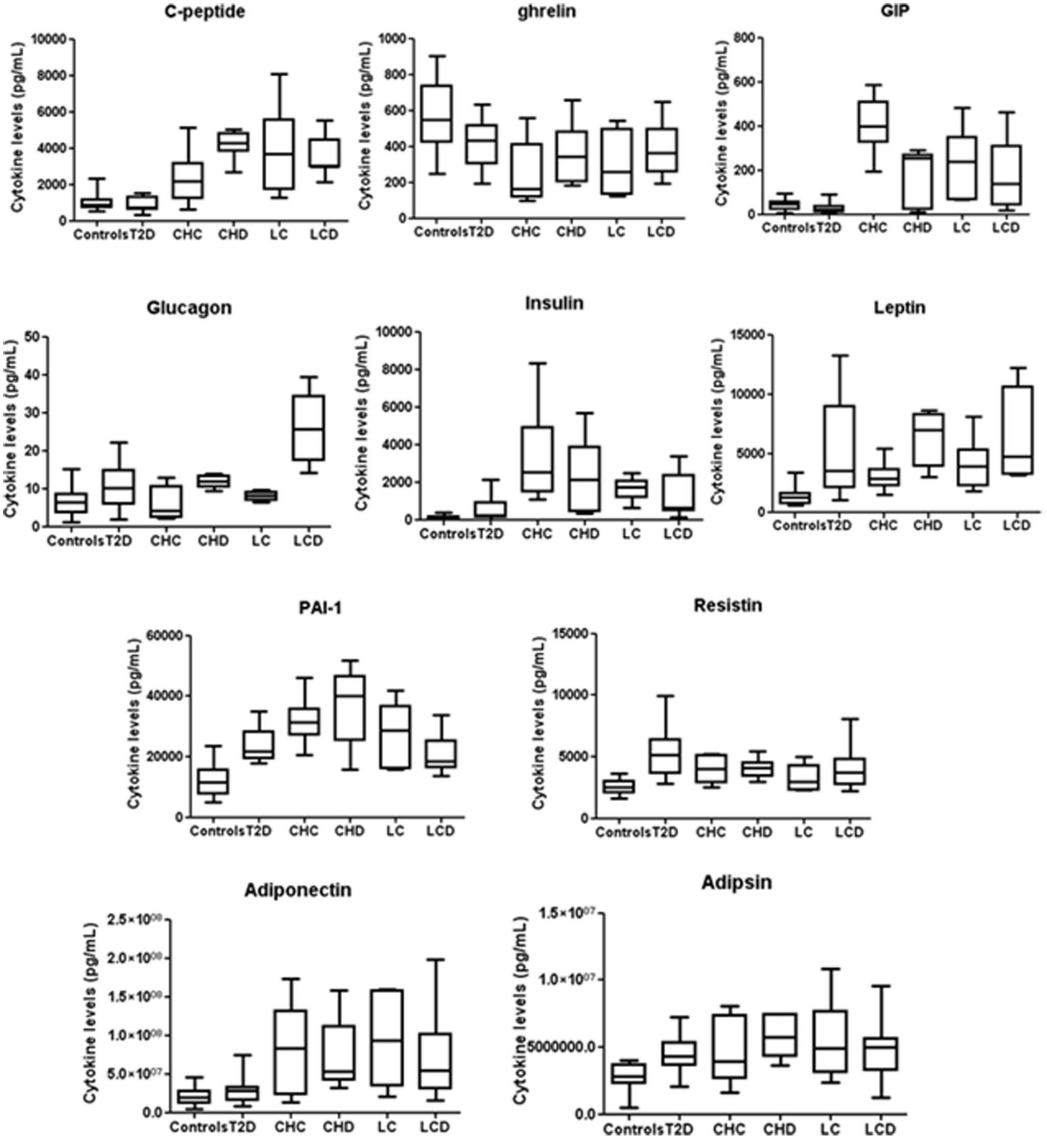
Significant cytokines in some patient groups. We report the significant molecule levels from controls and patients with type 2 diabetes (T2D), chronic hepatitis C (CHC), CHC-related cirrhosis (LC), CHC hepatitis and type 2 diabetes (CHD) and CHC-related cirrhosis and type 2 diabetes (LCD), evaluated by 10+2-Plex panel, are plotted with box-and-whisker graphs. The boxes extend from the 25th to the 75th percentile, and the line in the middle is the median. The error bars extend down to the lowest value and up to the highest.

In Figures 1–2 and in Table 2 we report the proteins that show different serum levels in the type 2 diabetes (T2D) patients respect to controls; data that were not statistically significant are not reported. Greater amounts of IL-1α, IL-2R, IL-12, IL-18, HGF, MIF, glucagon, insulin, leptin, PAI-1, resistin and adipsin and lower of ghrelin were secreted by T2D patients. The significantly increased serum levels of four pro-inflammatory interleukins (IL-1α, IL-2R, IL-12 and IL-18) are in agreement with already published data [15]–[16] that showed and confirmed how the inflammation course is more severe in diabetic patients than in non-diabetic subjects. Elevated HGF and MIF levels were indicated as markers of atherosclerotic and cardiomyopathic complications in T2D patients, respectively [17]–[18]. On the other hand, the increased levels of leptin, insulin, resistin and adipsin and the decreased levels of ghrelin are certainly correlated between them because adipsin is activated by insulin, whereas ghrelin and leptin exert antagonistic effects via their specific receptors. In fact, in hepatocytes ghrelin reduces and leptin augments insulin signal transduction [19]. Also, it has recently shown that the levels of resistin are correlated to those of leptin and that both these adipokines induce increased procoagulability in diabetes mellitus [20], [21]. Certainly, the elevated concentrations of glucagon in T2D patients depend from its role in dysregulated hepatic glucose production and abnormal glucose homeostasis [22]. Finally, since the platelet function is enhanced in T2D patients, the increased levels of PAI-1 (plasminogen activator inhibitor type 1) are considered to be a risk factor of cardiovascular diseases in these patients [23]. Since smoking status was known for T2D patients, we have subdivided them in two different subgroups, i.e. smokers and not smokers, and compared their cytokine levels to understand what proteins were related to cigarette smoking. The analysis showed that there were no statistically significant differences for the cytokines between the two subgroups of patients.

**Table 2:** Comparison of cytokine serum levels in all patients and healthy controls.

Cytokines	Controls vs T2D	Controls vs CHC	Controls vs CHD	Controls vs LC	Controls vs LCD
*21-Plex*					
IL-1α	*	**	***	***	***
IL-2R	*	*	**	**	***
IL-12	*	*	***	***	***
IL-18	*	*	***	**	***
CXCL1		**	**	**	**
CXCL9		*	***	***	***
HGF	*		**	**	**
β-NGF		**	**	***	***
MIF	*	*	***	***	***
*10-Plex*					
C-peptide		*	**	**	**
Ghrelin	*	**	*	*	*
GIP		**	*	*	*
Glucagon	*		*		**
Insulin	*	**	**	**	**
Leptin	**	*	**	**	**
PAI-1	*	**	**	**	**
Resistin	*	*	*	*	*
*2-Plex*					
Adiponectin		*	*	*	*
Adipsin	*	*	*	*	*

We report P-values (p) obtained for all significant molecules in patients with type 2 diabetes (T2D), chronic hepatitis C (CHC), CHC-related cirrhosis (LC), CHC hepatitis and type 2 diabetes (CHD) and CHC-related cirrhosis and type 2 diabetes (LCD), with respect to healthy control subjects using the nonparametric Mann-Whitney U test. In particular p<0.05 is indicated with *, p<0.01 with **, and p<0.001 with ***.

Then, we have correlated the serum levels of all the significant proteins in T2D patients with clinical/biochemical data by Pearson correlation coefficient (Table 3). In these patients, IL-18, resistin and MIF showed a significant correlation with BMI and glycemia levels (see Table 2). This result confirms these three proteins can be considered as predictors of inflammatory activation during the progression of T2D, as recently reported [21].

**Table 3:** Significant correlations between cytokines and clinical/biochemical data in all patients.

Disease	Cytokines	Clinical Data
**T2D**	IL-18, resistin, MIF	glycemia, BMI
**CHC**	IL-2R, MIF, β-NGF	transaminase
**CHD**	IL-18, C-peptide	glycemia, transaminasi, BMI
**LC**	CXCL1, CXCL9, HGF	albumin
**LCD**	Glucagon, HGF	glycemia, BMI, albumin

We report in patients with type 2 diabetes (T2D), chronic hepatitis C (CHC), CHC-related cirrhosis (LC), CHC hepatitis and type 2 diabetes (CHD) and CHC-related cirrhosis and type 2 diabetes (LCD) the cytokines and the clinical/biochemical data that correlate between them.

### Comparison between Patients with Chronic CHC Hepatitis and Healthy Donors

In CHC patients greater amounts of IL-1α, IL-2R, IL-12, IL-18, CXCL1, CXCL9, MIF, β-NGF, C-peptide, GIP, insulin, leptin, PAI-1, resistin, adiponectin and adipsin and lower of ghrelin were secreted with respect to healthy controls (Figures 1–2 and Table 2).

The increased serum levels of IL-1α, IL-2R, CXCL1, CXCL9, MIF and β-NGF agree with our recently published data [13] since they are all pro-inflammatory cytokines involved in the immune response to CHC and drive liver damage. Moreover, during inflammation both IL-12 and IL-18 are produced by macrophages, work together in inducing cell-mediated immunity and stimulate the production of CXCL9 [24].

Also in literature, increased levels of leptin, resistin and adiponectin as well as of PAI-1 have been found in CHC patients and they have been correlated to the severity of fibrosis and, hence, indicated as marker in detecting HCC when the carcinogenesis is affected by CHC infection [25]–[26].

In general, C-peptide serves as an important link between the insulin chains and facilitates the efficient assembly, the folding and the processing of insulin in the endoplasmic reticulum. Higher C-peptide and insulin levels in CHC patients were already reported supporting the hypothesis that insulin resistance may contribute to fibrotic progression in CHC infection [5]. The secretion of adipsin, as reported above, is activated by insulin [19] whereas GIP (called gastrointestinal inhibitory peptide) induces insulin secretion [27]. Hence the increased levels of GIP, adipsin and insulin as well as of leptin, that augments insulin signal transduction, are mainly correlated not only between them but also with the decreased levels of ghrelin that has been indicated as marker of the liver fibrosis progression [19].

However, no data have been found in literature related to GIP and adipsin evaluations in CHC patients and our integrated analysis suggests, for the first time, a role for GIP and adipsin in CHC-related chronic inflammation.

From the correlation of the serum levels of all the significant proteins in CHC group with clinical/biochemical data, it is resulted that IL-2R, MIF and β-NGF showed a positive correlation coefficient with the transaminase values (Table 3), which were higher in these patients than in healthy controls. This confirms the role of these proteins as index of immune activation according to our recently published data [13].

### Comparison between Patients with Type 2 Diabetes and those with Chronic CHC Hepatitis

In both T2D and CHC patients, IL-1α, IL-2R, IL-12, IL-18, MIF, insulin, leptin, PAI-1, resistin and adipsin are increased whereas ghrelin is decreased with respect to healthy controls (Table 2 and Figures 1–2). We used T-test to compare the serum levels of these proteins evaluated in different patients groups. In particular, IL-2R, IL-12, IL-18, MIF, resistin and adipsin have similar concentrations in both diseases. In CHC patients ghrelin and leptin levels are lower than those with T2D (with p-value <0.05) whereas IL-1α, insulin and PAI-1 levels are higher (with p-value <0.05).

Moreover, CXCL1, CXCL9, β-NGF, C-peptide, GIP and adiponectin are higher only in CHC patients in agreement with previous studies in which these molecules were found elevated only in diabetic patients with complications (nephropathy, retinopathy and atherosclerotic lesions) [28]–[32].

On the other hand, HGF and glucagon are higher only in T2D patients and this confirms that glucagon and HGF play a specific role in glucose production and homeostasis [23] and in the progression of chronic inflammation to liver cirrhosis and cancer [13], respectively.

### Patients with Chronic CHC Hepatitis and Type 2 Diabetes (CHD)

In CHD patients greater amounts of IL-1α, IL-2R, IL-12, IL-18, CXCL1, CXCL9, HGF, MIF, β-NGF, C-peptide, GIP, glucagon, insulin, leptin, PAI-1, resistin, adiponectin and adipsin and lower of ghrelin were secreted than in healthy controls (Figures 1–2 and Table 2). Since in CHD patients there are increased levels of the same molecules resulted significant in type 2 diabetes and/or CHC patients, we compared by T-test the serum concentrations in CHD patients and those evaluated in patients having only type 2 diabetes or CHC to understand what cytokines change or unchange in presence of both diseases.

The levels of MIF, insulin, ghrelin, leptin, glucagon, resistin and adipsin are similar in type 2 diabetes and CHD patients whereas IL-1α, IL-2R, IL-12, IL-18, HGF, and PAI-1 are higher in CHD patients than in type 2 diabetes patients (with p-value <0.01).

On the other hand, the levels of CXCL1, β-NGF, resistin, adiponectin, adipsin and PAI-1 are similar in CHC and CHD patients whereas IL-1α, IL-2R, IL-12, IL-18, leptin, C-peptide, ghrelin and CXCL9 are higher in CHD patients than in CHC patients and GIP is lower in CHD patients than in CHC patients (with p-value <0.01).

Overall these data highlight that the simultaneous presence of CHC and type 2 diabetes induces an evident increase of the following proinflammatory cytokines: IL-1α, IL-2R, IL-12, IL-18, CXCL9, MIF and HGF. In fact, in CHD patients their levels are higher than in type 2 diabetes or CHC patients suggesting an associative effect of two diseases. In particular, in comparison to C-peptide, ghrelin, GIP, insulin and adiponectin, it is worth noting that CXCL9, HGF and glucagon levels are increased in CHD patients even if they were resulted significant in patients affected by one only disease.

As in the case of T2D and CHC patients, we have correlated the serum levels of all the significant proteins in CHD group with clinical/biochemical data. In these patients both IL-18 and C-peptide showed a significant positive correlation with transaminases and BMI values (Table 3). Moreover, IL-18 correlated also with glycemia as in type 2 diabetes patients. Certainly, these data are interesting since IL-18 is increased in both CHC and type 2 diabetes patients whereas C-peptide is increased only in CHC patients. Therefore, an increased concentration of C-peptide associated with elevated serum levels of transaminases can be used as index of viral infection in diabetic hyperglycemic patients.

### Patients with CHC-related Cirrhosis (LC)

Greater amounts of IL-1α, IL-2R, IL-12, IL-18, CXCL1, CXCL9, MIF, β-NGF, HGF, C-peptide, GIP, insulin, PAI-1, adiponectin, leptin, resistin and adipsin and lower of ghrelin were secreted by LC patients respect to controls (Figures 1–2). These results have evidenced that high circulating levels of these molecules are correlated to the presence of fasting glucose in cirrhotic patients due to reduced liver function and altered hepatic glucose metabolism in agreement to previous papers [33]–[39]. However, it is important to underline that the same molecules increased in CHC and LC patients with the sole exception of HGF that can be used for predicting the progression to HCC in patients with CHC-related liver disease and low albumin.

**Figure 3:**
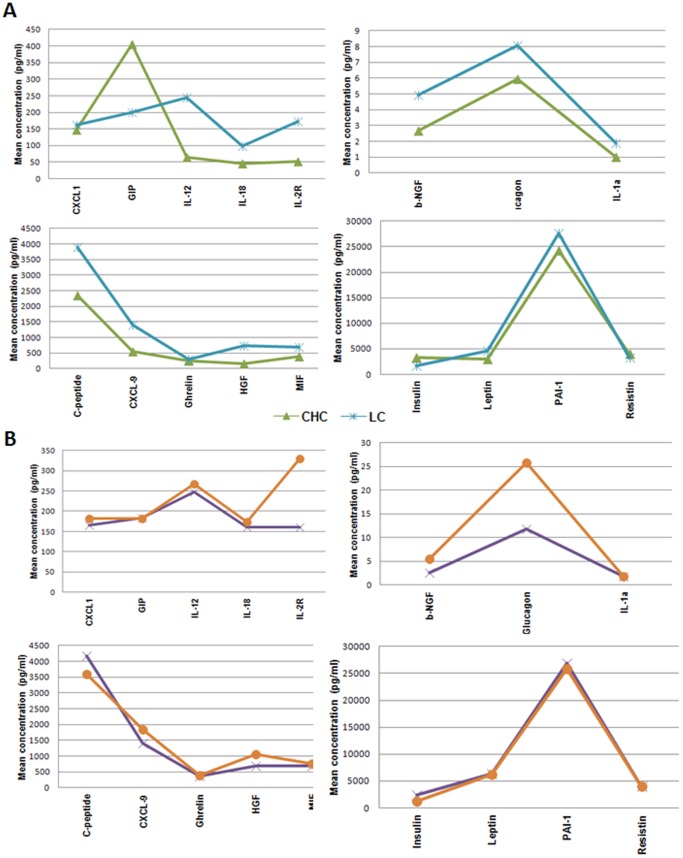
Mean concentrations of significant cytokines. We report the mean levels of the molecules evaluated in patients with chronic hepatitis C (CHC) and CHC-related cirrhosis (LC) (A), and with CHC hepatitis and type 2 diabetes (CHD) and CHC-related cirrhosis and type 2 diabetes (LCD) (B). The legends evidence the different colors used for the analyzed groups.

We have used the T-test to compare the levels of all the significant proteins in CHC as well as LC patients. Insulin, PAI-1, adiponectin, resistin and adipsin levels are similar in both groups whereas GIP is lower in LCs than in CHC patients (with p-value <0.05) and C-peptide, IL-1α, IL-2R, IL-12, IL-18, CXCL9, MIF, β-NGF, HGF and leptin are higher in LCs than in CHC patients (with p-value <0.05). These data suggest that these pro-inflammatory cytokines increase during the chronic inflammation leading to LC and, then, to cancer. Since it has been reported in the literature that T2D can be associated with HCC in CHC patients without cirrhosis [1], [4], [9], we have also compared by T-test the levels of significant molecules in CHD and LC patients to separate cytokines in two groups (similar or different) in order to use them as specific markers of the two different processes. In particular, only three molecules behave differently in CHD and LC patients: β-NGF is higher in LC whereas glucagon and IL-18 are higher in CHD (with p-value <0.05) (Figure 3). This suggests that β-NGF is a pro-inflammatory protein and index of inflammatory process related to chronic infection leading to cirrhosis and then to cancer, whereas glucagon and IL-18 are mainly due to diabetes that occurs as part of the metabolic syndrome with an increased risk of HCC. Moreover, in agreement with our recent results [13], the levels of CXCL1, CXCL9 and HFG in patients with LC showed a significant positive correlation among them, and a negative correlation with the albumin values (Table 3), which are lower in these patients in respect to controls.

### Patients with CHC-related Cirrhosis and Type 2 Diabetes (LCD)

Greater amounts of IL-1α, IL-2R, IL-12, IL-18, CXCL1, CXCL9, MIF, β-NGF, HGF, C-peptide, GIP, insulin, PAI-1, adiponectin, leptin, resistin, ghrelin, and adipsin and lower amounts of ghrelin were found in LCD patients compared with healthy donors (Figures 1–2 and Table 2). It is also evident that the same molecules are significant in LC and LCD patients. The only difference is represented from glucagon (with p-value <0.05) that is significant in LCD patients and not in LC patients. Concerning the levels of all the significant molecules we can underline that they are similar in two groups with the exception of IL-2R, IL-18 and glucagon that are higher in LCD patients. This indicates that the levels of the three molecules are due to the presence of type 2 diabetes in LC patients. Finally, from the correlation of the serum levels of all the significant proteins in LCD group with clinical/biochemical data (Table 3), we observe that glucagon and HGF show a significant positive correlation among them and with glycemia and BMI values while correlate negatively with albumin values, which are lower in these patients compared to controls.

## Discussion

In this paper we report a simultaneous and comparative analysis of serum levels of a large panel of cytokines, chemokines, adipokines and growth factors in patients with type 2 diabetes, CHC, CHC-related cirrhosis, CHC and type 2 diabetes and CHC-related cirrhosis and type 2 diabetes by BioPlex assay. Our interest for these diseases depends from the fact that Southern Italy shows the highest rates of type2 diabetes/obesity [40] and, also, of liver cancer for Europe, mainly related to infection with hepatitis viruses (i.e. CHC) [41].

A general view of the results in Table 2, shows that β-NGF, CXCL1, CXCL9, C-peptide, GIP and adiponectin resulted up-expressed in all the patient groups except in those with T2D suggesting that they can be associated to the chronic CHC infection leading to fibrotic, cirrhotic and cancer progression, also in presence of type 2 diabetes (CHD and LCD). In fact, β-NGF is involved in cancer growth and metastasis and it has also been detected in diseased liver tissues [11], CXCL1 and CXCL9 have chemotactic activities and roles in angiogenesis, inflammation and tumor genesis [10], adiponectin is expressed on monocytes and macrophages that mediate the stimulation of protein kinase activated by AMP, the oxidation of fatty acids and the uptake of glucose [42], whereas C-peptide is implicated in insulin resistance [5] and GIP induces insulin secretion [27].

Since type 2 diabetes has been recognized as a cofactor that can modify the course of chronic CHC infection and can be used as an independent predictor of HCC [8], we have compared the significant molecules in patients with T2D and CHC. In details, we have evidenced that: i) ghrelin and leptin levels are lower in CHC patients than in those with T2D, ii) IL-1α, insulin and PAI-1 levels are higher in CHC patients than in those with T2D, iii) CXCL1, CXCL9, β-NGF, C-peptide, GIP and adiponectin are higher only in CHC patients and iv) HGF and glucagon are higher only in T2D patients.

**Figure 4:**
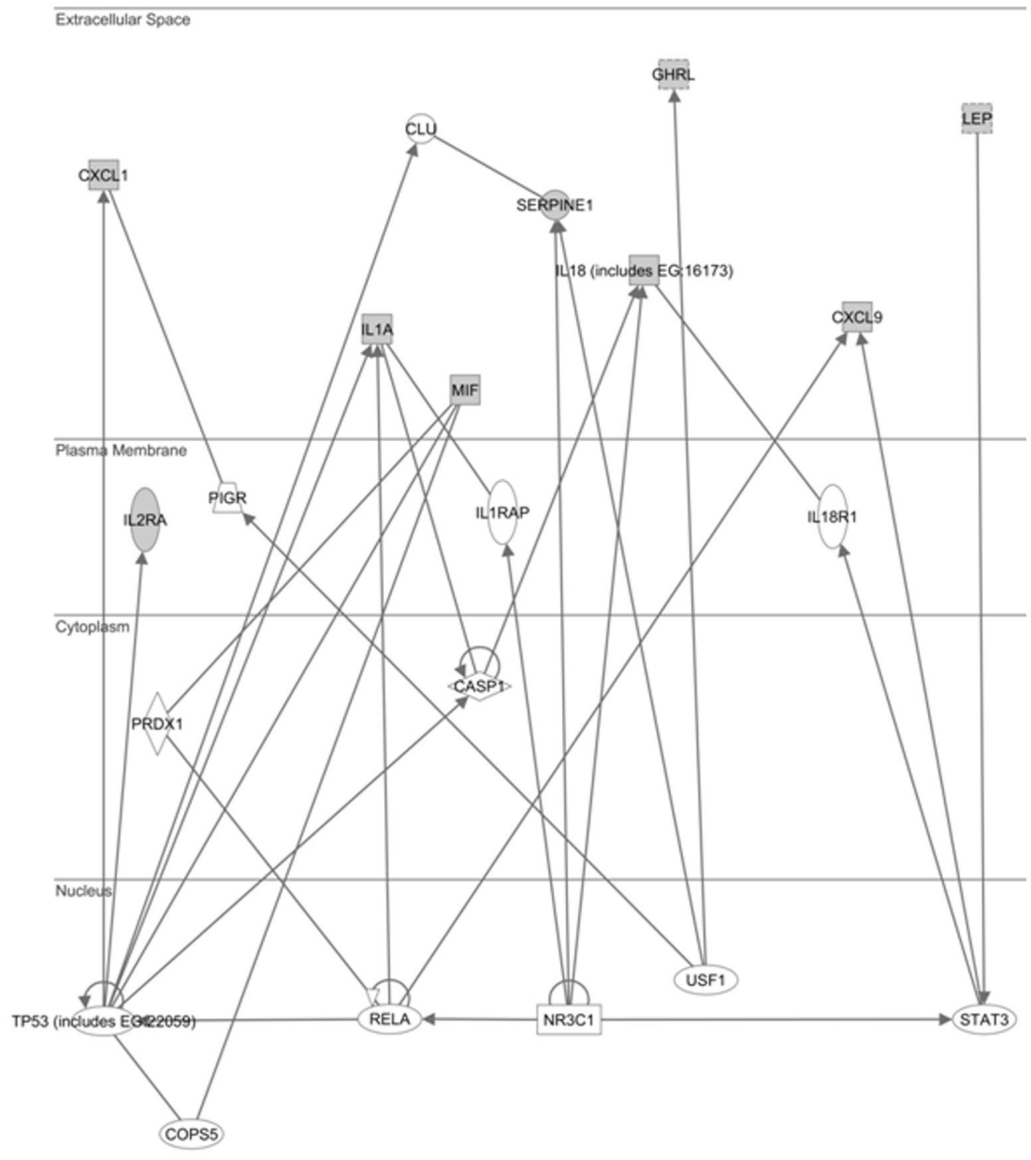
Ingenuity Pathway Analysis (IPA) of significant molecules. The graph shows the closely associated network for significant cytokines, reported also in Table 2, (evidenced with grey symbols) and other molecules (evidenced with white symbols) obtained by IPA software.

All these molecules were analysed by Ingenuity Pathway Analysis 7.1 (Ingenuity Systems, Inc., Redwood City, CA, USA) that created a network on the basis of associated functions and data mining from experimental studies reported in literature (Figure 4 and Table S1). This graph presents five hub genes such as STAT3 (signal transducer and activator of transcription), NR3C1 (nuclear receptor subfamily 3, group C, member 1), NF-kB (called also RELA), TP53 (tumor p53) and USF1 (upstream stimulatory factor 1). In particular, STAT3 is induced from the leptin [43] and has a role in suppressing IFN induction of STAT1-dependent inflammatory genes, such as CXCL9 [44]. Therefore, we can hypothesize that in T2D patients the higher level of leptin induces the STAT3 increased expression and the related CXCL9 suppression. On the other hand, NR3C1 is the receptor to which cortisol and other glucocorticoids bind and interacts with STAT3 and RELA [45]. The network reported in Figure 4 shows that RELA modulates the expression of TP53, MIF, CXCL9 and IL-1α whereas TP53 that of CXCL1, IL2R, IL-1α, MIF, PAI-1 (called also SERPINE1) and IL18. In literature it is reported that two RELA and TP53 genes are up-expressed in CHC and liver fibrosis [46] and this can explain because CXCL1, IL-1α IL18 and PAI-1 as well as CXCL9 are higher in CHC patients. As regards USF1, it is involved in the regulation of numerous genes of glucose and lipid metabolism as well as hepatic metabolism. USF1 resulted down-expressed during the progression of liver fibrosis in CHC patients [47] and this explains the lower ghrelin expression in CHC patients in respect to those with T2D.

Moreover, it is worth of note that HGF has been found normal only in CHC patients. This growth factor stimulates mitogenic, motogenic (including metastogenesis) and morphogenic effects on epithelial and endothelial cells via its receptor, proto-oncogene Met. The activation of Met by HGF binding is linked to cell growth and survival, through activation of both the PI3-kinase/PDK/Akt and the Ras/Raf/MEK/ERK pathways, and to cell mobility and cytoskeletal organization via activation of the Rho-GTPases, Rho, Rac and CDC42. HGF and Met have been associated with progression, invasiveness and metastasis in a number of cancer types and for these reasons their signaling is a major target for cancer therapeutics development [48]. Hence, HGF increased levels in all patient groups except in CHC confirm and suggest that this protein can be used as index for predicting, in patients with T2D, the progression of chronic inflammation to cancer [13].

The analysis of how molecule levels change during the progression to more advanced stages, showed that IL-1α, IL-2R, IL-12, IL-18, CXCL9, MIF and HGF are higher in CHD, LC and LCD than in patients with only type 2 diabetes or CHC suggesting that these proteins are associative markers of these two diseases and implicated in their complicance and progression.

Furthermore, we have compared cytokinomes of LC and CHD patients to realize what molecules were significantly different in these two stages because in literature it has been reported that patients with chronic CHC infection and type 2 diabetes without liver cirrhosis can evolve into hepatocarcinogenesis [8]–[9]. In details, β-NGF is higher in LC patients whereas glucagon and IL-18 are higher in CHD patients (Figure 3). Hence, data evidence that these three molecules can be associated to two different stages of the pathological processes (CHD and LC) that may lead to cancer. Therefore, we suggest that β-NGF can be helpful as index of that chronic infection stage able to evolve into cirrhosis and, then, HCC while glucagon and IL-18 can be index of the diabetes association that occurs as part of the metabolic syndrome leading to HCC.

In overall the obtained results evidence that the serum levels of some cytokines, chemokines, adipokines and growth factors can be useful for the clinical monitoring of patients because they give specific information in regard to the progression from CHC and LC and to that from CHD to LCD. In fact, we have evaluated the ratio between the mean concentrations of significant molecules in LC versus CHC patients and in LCD vs CHD patients (see Table S2) and in Figures 3A and B the mean concentrations of all the significant molecules are reported in order to compare quickly the different cytokinome profiles in the disease progression of these patients. In details, the levels of GIP are lower in LC patients than in CHC patients whereas those of C-peptide, IL-1α, IL-2R, IL-12, IL-18, CXCL9, MIF, β-NGF, HGF and leptin are higher in LC patients than in CHC patients. This suggests that these proteins can be used to monitor and follow the CHC patients and to understand whether they are leading to LC. Comparing CHD and LCD patients we can observe that four proteins, i.e. β-NGF, glucagon, IL-2R and HGF, present in LCD patients concentrations significantly higher than those with CHD. Therefore we suggest that the monitoring of these four molecules can be useful to improve the prognosis of CHD patients.

At present our data are only indicative of HCC risk only showing that cytokines are up-expressed in CHD, LCD and LC as stage before cancer. However, we have already planned to collect sera of HCC patients with or without T2D to identify the cytokinome profile of these patients and explain what cytokines can be marker of HCC risk.

Moreover, we have correlated the serum levels of all the significant proteins in each group with clinical/biochemical data by Pearson correlation coefficient (Table 3). This analysis has evidenced that: i) in T2D patients IL-18, resistin and MIF correlated positively with glycemia and BMI levels, ii) in CHC patients IL-2R, MIF and β-NGF correlated positively with transaminase values, iii) in CHD patients IL-18 and C-peptide correlated positively with transaminase, BMI and glycemia values, iv) in LC patients CXCL1, CXCL9 and HFG correlated negatively with albumin values and v) in LCD patients glucagon and HGF correlated positively with BMI and glycemia values and negatively with albumin values. All the results have suggested the possibility to use mini-panels composed from cytokines and biochemical data for discriminating the various stages of these diseases.

To evaluate the possible association between the serum levels of all the significant proteins in each group and smoking status, hypertension, BMI, and hypercholesterolemia, we performed a multivariate regression analysis using the cytokines as dependent variables and these parameters as independent variables (Table 4). In particular, the smoking status was known only for T2D patients. As evidenced from odds ratios (OR) higher than 1, BMI values resulted positively related to high levels of IL-8, resistin and MIF in T2D patients, of IL-18 and C-peptide in CHD patients and of glucagon and HGF in LCD patients. On the other hand, cytokine levels were not associated with hypertension and hypercholesterolemia in all the groups as well as with the smoking status in T2D patients (*data not shown*).

**Table 4:** Multivariate regression analysis using the cytokines as dependent variables and smoking status, hypertension, BMI, and hypercholesterolemia as independent variables.

Cytokines	OR (95% CI)				
	T2D	CHC	CHD	LC	LCD
*21-Plex*					
IL-1α	0.66 (0.32–1.12)	0.72 (0.44–1.06)	0.69 (0.39–1.02)	0.88 (0.69–1.11)	0.79 (0.49–1.08)
IL-2R	0.65 (0.21–1.01)	0.79 (0.48–1.04)	0.72 (0.42–1.01)	0.85 (0.48–1.10)	0.81 (0.55–1.10)
IL-12	0.74 (0.50–1.05)	0.78 (0.52–1.01)	0.65 (0.49–1.11)	0.77 (0.48–1.05)	0.80 (0.58–1.09)
IL-18	**5.36 (3.51–7.65)**	0.81 (0.56–1.15)	**4.51 (3.95–5.23)**	0.79 (0.45–1.21)	1.01 (0.89–1.20)
CXCL1		0.77 (0.42–1.05)	1.01 (0.72–1.41)	0.94 (0.55–1.29)	0.95 (0.78–1.20)
CXCL9		0.81 (0.49–1.07)	0.99 (0.58–1.09)	0.95 (0.51–1.28)	0.91 (0.79–1.13)
HGF	1.06 (0.52–1.55)		1.01 (0.71–1.44)	0.88 (0.71–1.15)	**3.45 (2.74–4.76)**
β-NGF		0.74 (0.47–1.08)	0.79 (0.50–1.13)	0.79 (0.50–1.09)	0.89 (0.71–1.10)
MIF	**2.36 (1.91–4.01)**	0.80 (0.59–1.04)	0.81 (0.57–1.11)	0.90 (0.68–1.12)	0.94 (0.75–1.12)
*10-Plex*					
C-peptide		0.69 (0.41–1.00)	**1.86 (1.55–3.92)**	0.87 (0.75–1.13)	1.00 (0.78–1.22)
Ghrelin	0.71 (0.44–1.10)	0.78(0.49–1.09)	0.80 (0.64–1.00)	0.93 (0.73–1.22)	0.92 (0.79–1.10)
GIP		0.75 (0.44–1.10)	0.78 (0.52–1.09)	0.76 (0.59–1.12)	0.89 (0.70–1.08)
Glucagon	0.73 (0.41–1.07)		0.99 (0.59–1.14)		**2.77 (1.98–3.89)**
Insulin	0.92 (0.56–1.45)	0.74 (0.45–1.05)	1.01 (0.79–1.23)	0.89 (0.66–1.11)	0.88 (0.75–1.06)
Leptin	0.65 (0.43–0.99)	0.84 (0.50–1.10)	1.00 (0.77–1.19)	0.91 (0.76–1.21)	0.89 (0.66–1.09)
PAI-1	0.44 (0.21–1.01)	0.90 (0.74–1.11)	1.03 (0.75–1.30)	0.79 (0.54–1.13)	0.95 (0.79–1.19)
Resistin	**1.99 (1.52–3.21)**	0.71 (0.52–1.02)	1.05 (0.75–1.28)	0.85 (0.57–1.09)	0.99 (0.78–1.21)
*2-Plex*					
Adiponectin		0.69 (0.40–0.99)	0.98 (0.79–1.19)	0.79 (0.60–1.07)	0.70 (0.51–1.00)
Adipsin	0.55 (0.38–1.04)	0.73 (0.50–1.03)	1.01 (0.68–1.19)	0.84 (0.51–1.05)	0.72 (0.49–1.08)

We report odds ratios (OR) and 95% confidence intervals (95% CI) evaluated for association between each significant cytokine (shown in Table 2) and BMI in patients with type 2 diabetes (T2D), chronic hepatitis C (CHC), CHC hepatitis and type 2 diabetes (CHD), CHC-related cirrhosis (LC), and CHC-related cirrhosis and type 2 diabetes (LCD). OR being positively associated (higher than 1) to cytokines are evidenced in bold.

In overall, our observations demonstrate that an integrated approach (multiplex bead-based assays) is much more powerful than isolated measurements to evaluate specific stages of these two complex pathologies (type 2 diabetes and chronic CHC hepatitis) alone or when they are concomitant in a patient. Reproducibility (coefficient of variance), degree of multiplexing (plex level), reliability (publication history) and higher sensitivity are the best feature of this assay. The interconnected, vital extracellular signaling networks of cytokines, are at least as complex as intracellular signaling pathways. Cellular signals when released into the extracellular milieu are strongly diluted and, hence, the detection of multiple cytokines is necessary to get a more global view of these signals. The less that is known about a particular physiologic or pathologic mechanism directed by cytokine signaling, the greater the need to use multiplexed cytokine detection. However, when we are monitoring changes in levels in certain disease conditions, what is important is to have a correct ratio between the cytokines expressed at high concentration compared to those expressed at very low concentrations to the limits of detectability in experiments which follow these changes dynamically over time. The correct ratio is much more important than single measured quantities because it represents the balance of population among the signaling molecules, also it makes the results more significant because internally consistent. Therefore, the multiplex assay has emerged as an accurate, simple, specific, noninvasive, reproducible and less expensive method that, in future, could be included in routine clinical practice to monitor the association of type 2 diabetes and/or CHC to liver cirrhosis, and to improve the prognosis of these diseases. Work is already in progress to collect in specialized databanks as much data is possible on cytokinomes determined by multiplexing as well as clinical/biochemical data to distinguish and evaluate quantitatively the pathological patterns of these patients by a neural network approach [49]. Of course the presence of protein chips able to evaluate comparatively the amounts of a high number of cytokines is an important prerequisite to improve the discriminating ability of this approach as well as the decision-making ability of the neural networks.

In conclusion our working hypothesis was i) to understand if the cytokinome profiles can be used to distinguish the patients with only CHC or T2D or with both diseases, ii) to identify what cytokines present concentrations similar or different in CHD and LC patients because in the literature it has reported that T2D can be associated with HCC in HCV patients without cirrhosis iii) to identify what cytokines can be useful for the clinical monitoring of patients in the progression from CHC and LC and to that from CHD to LCD and iv) to define the prognostic mini-panels for these diseases based on biochemical data and cytokines. The results obtained in our paper have evidenced that some cytokines are significantly up-regulated in patients with only CHC or T2D whereas other cytokines increase when both the diseases are present. Moreover, from the comparison between the levels of significant molecules in CHD and LC patients, it is resulted that three molecules present different behavior in these patients: β-NGF is higher in LC whereas glucagon and IL-18 are higher in CHD. Therefore we suggest that β-NGF is a pro-inflammatory protein and index of inflammatory process related to chronic infection leading to cirrhosis and, then, to cancer whereas glucagon and IL-18 are due mainly to the diabetes and occurs as part of the metabolic syndrome that can increase the risk of HCC. Also our results have evidenced what cytokines change in the progression from CHC and LC and to that from CHD to LCD. Finally our studies have demonstrated that some molecules have significant correlations and associations with clinical/biochemical data (see Tables 3 and 4), and defined mini-panels that can be used as specific markers for the different disease staging.

## Supporting Information

Table S1 **List of molecules involved in the network reported in** **figure 4** **.** For each gene the table shows the symbol, Entrez Gene Name, Cellular Location and the type.
(DOC)

Table S2 **Ratio between the mean concentrations of significant molecules in LC versus CHC patients and in LCD vs CHD patients**. We have evidenced in bold the ratio >1.5
(DOC)

## References

[1] Lai MS, Hsieh MS, Chiu YH, Chen TH (2006). Type 2 diabetes and hepatocellular carcinoma: A cohort study in high prevalence area of hepatitis virus infection.. Hepatology.

[2] Veldt BJ, Chen W, Heathcote EJ, Wedemeyer H, Reichen J (2008). Increased risk of hepatocellular carcinoma among patients with hepatitis C cirrhosis and diabetes mellitus.. Hepatology.

[3] Chen CL, Yang HI, Yang WS, Liu CJ, Chen PJ (2008). Metabolic factors and risk of hepatocellular carcinoma by chronic hepatitis B/C infection: a follow-up study in Taiwan.. Gastroenterology.

[4] Hung CH, Lee CM, Chen CH, Hu TH, Jiang SR (2009). Association of inflammatory and anti-inflammatory cytokines with insulin resistance in chronic hepatitis C. Liver Int.

[5] Hui JM, Sud A, Farrell GC, Bandara P, Byth K (2003). Insulin resistance is associated with chronic hepatitis C virus infection and fibrosis progression.. Gastroenterology.

[6] Huang JF, Dai CY, Hwang SJ, Ho CK, Hsiao PJ (2007). Hepatitis C viremia increases the association with type 2 diabetes mellitus in a hepatitis B and C endemic area: an epidemiological link with virological implication.. Am J Gastroenterol.

[7] Castello G, Costantini S, Scala S (2010). Targeting the inflammation in CHC-associated hepatocellular carcinoma: a role in the prevention and treatment.. J Transl Med.

[8] Hung CH, Lee CM, Wang JH, Hu TH, Chen CH (2011). Impact of diabetes mellitus on incidence of hepatocellular carcinoma in chronic hepatitis C patients treated with interferon-based antiviral therapy.. Int J Cancer.

[9] Paradis V, Zalinski S, Chelbi E, Guedj N, Degos F (2009). Hepatocellular carcinomas in patients with metabolic syndrome often develop without significant liver fibrosis: a pathological analysis.. Hepatology.

[10] Capone F, Costantini S, Guerriero E, Calemma R, Napolitano M (2010). Serum cytokine levels in patients with hepatocellular carcinoma.. Eur Cyt Net.

[11] Costantini S, Autiero I, Colonna G (2008). On new challenge for the Bioinformatics.. Bioinformation.

[12] Costantini S, Castello G, Colonna G (2010). Human Cytokinome: a new challenge for systems biology.. Bioinformation.

[13] Costantini S, Capone F, Guerriero E, Maio P, Colonna G (2010). Serum cytokine levels as putative prognostic markers in the progression of chronic CHC hepatitis leading to cirrhosis.. Eur Cyt Netw.

[14] Standard of Medical Care in Diabetes (2009). Diabetes Care.

[15] Chao PC, Huang CN, Hsu CC, Yin MC, Guo YR (2010). Association of dietary AGEs with circulating AGEs, glycated LDL, IL-1α and MCP-1 levels in type 2 diabetic patients.. Eur J Nutr.

[16] Yao K, Lu H, Huang R, Zhang S, Hong X (2011). Changes of dendritic cells and fractalkine in type 2 diabetic patients with unstable angina pectoris: a preliminary report.. Cardiovasc Diabetol.

[17] Yu XY, Chen HM, Liang JL, Lin QX, Tan HH (2011). Hyperglycemic myocardial damage is mediated by proinflammatory cytokine: macrophage migration inhibitory factor.. PLoS One.

[18] Anan F, Masaki T, Jikumaru K, Iwao T, Eshima N (2010). Hepatocyte growth factor is a significant risk factor for white matter lesions in Japanese type 2 diabetic patients.. Eur J Clin Invest.

[19] Fantuzzi G (2005). Adipose tissue, adipokines, and inflammation.. Journal of Allergy and Clinical Immunology.

[20] Bobbert P, Eisenreich A, Weithäuser A, Schultheiss HP, Rauch U 2(011) Leptin and resistin induce increased procoagulability in diabetes mellitus.. Cytokine.

[21] Netea MG, Joosten LAB, Lewis E, Jensen DR, Voshol PJ (2006). Deficiency of interleukin-18 in mice leads to hyperphagia, obesity and insulin resistance.. Nature Medicine.

[22] D’Alessio D (2011). The role of dysregulated glucagon secretion in type 2 diabetes.. Diabetes Obes Metab.

[23] Konya H, Hasegawa Y, Hamaguchi T, Satani K, Umehara A (2010). Effects of gliclazide on platelet aggregation and the plasminogen activator inhibitor type 1 level in patients with type 2 diabetes mellitus.. Metabolism.

[24] Coma G, Peña R, Blanco J, Rosell A, Borras FE (2006). Treatment of monocytes with interleukin (IL)-12 plus IL-18 stimulates survival, differentiation and the production of CXC chemokine ligands CXCL8, CXCL9 and CXCL10.. Clin Exp Immunol.

[25] Divella R, Lacalamita R, Tommasi S, Coviello M, Daniele A (2008). PAI-1, t-PA and circulating hTERT DNA as related to virus infection in liver carcinogenesis.. Anticancer Res.

[26] Marra F, Bertalani C (2009). Adipokines in liver diseases.. Hepatology.

[27] Thorens B (1995). Glucagon-like peptide-1 and control of insulin secretion.. Diabète & métabolisme.

[28] Alfadda AA, Alzoghaibi MA (2008). Circulatory neutrophil chemokines in statin-treated diabetic patients.. Saudi Med J.

[29] Osei K, Falko JM, O’Dorisio TM, Fields PG, Bossetti B (1986). Gastric inhibitory polypeptide responses and glucose turnover rates after natural meals in type II diabetic patients.. J Clin Endocrinol Metab.

[30] Wong CK, Ho AW, Tong PC, Yeung CY, Kong AP (2007). Aberrant activation profile of cytokines and mitogen-activated protein kinases in type 2 diabetic patients with nephropathy.. Clin Exp Immunol.

[31] Park KS, Kim SS, Kim JC, Kim HC, Im YS (2008). Serum and tear levels of nerve growth factor in diabetic retinopathy patients Am J Ophthalmol.

[32] Kim ST, Kim BJ, Lim DM, Song IG, Jung JH (2011). Basal C-peptide Level as a Surrogate Marker of Subclinical Atherosclerosis in Type 2 Diabetic Patients.. Diabetes Metab J.

[33] Montoliu C, Piedrafita B, Serra MA, del Olmo JA, Urios A (2009). IL-6 and IL-18 in Blood May Discriminate Cirrhotic Patients With and Without Minimal Hepatic Encephalopathy.. Journal of Clinical Gastroenterology.

[34] Sharma A, Chakraborti A, Das A, Dhiman RK, Chawla Y (2009). Elevation of interleukin-18 in chronic hepatitis C: implications for hepatitis C virus pathogenesis.. Immunology.

[35] Kalaitzaki E, Bosaeus I, Öhman L, Björnsson E (2007). Altered postprandial glucose, insulin, leptin, and ghrelin in liver cirrhosis: correlations with energy intake and resting energy expenditure.. American Journal of Clinical Nutrition.

[36] Kruszynska YT, Ghatei MA, Bloom SR, McIntyre N (1995). Insulin secretion and plasma levels of glucose-dependent insulinotropic peptide and glucagon-like peptide 1 [7–36 amide] after oral glucose in cirrhosis.. Hepatology.

[37] Raya-Sánchez JM, González-Reimers E, Rodríguez-Martín JM, Santolaria-Fernández F, Molina-Pérez M (1998). Coagulation inhibitors in alcoholic liver cirrhosis.. Alcohol.

[38] Tietge UJF, Böker KHW, Manns MP, Bahr MJ (2004). Elevated circulating adiponectin levels in liver cirrhosis are associated with reduced liver function and altered hepatic hemodynamics.. American Journal of Physiology.

[39] Bahr MJ, Ockenga J, Böker KHW, Manns MP, Tietge UJF (2006). Elevated resistin levels in cirrhosis are associated with the proinflammatory state and altered hepatic glucose metabolism but not with insulin resistance.. American Journal of Physiology.

[40] Classen JB (2011). Italian pediatric data support hypothesis that simultaneous epidemics of type 1 diabetes and type 2 diabetes/metabolic syndrome/obesity are polar opposite responses (i.e., symptoms) to a primary inflammatory condition.. J Pediatr Endocrinol Metab.

[41] Fusco M, Girardi E, Piselli P, Palombino R, Polesel J (2008). Epidemiology of viral hepatitis infections in an area of southern Italy with high incidence rates of liver cancer.. Eur J Cancer.

[42] Kadowaki T, Yamauchi T (2005). Adiponectin and adiponectin receptors.. Endocr Rev.

[43] Carvalheira JBC, Siloto RMP, Ignacchitti I, Brenelli SL, Carvalho CRO (2001). Insulin modulates leptin-induced STAT3 activation in rat hypothalamus.. Febs Letters.

[44] Ho HH, Ivashkiv LB (2006). Role of STAT3 in Type I Interferon Responses.. JBC.

[45] Hollenberg SM, Weinberger C, Ong ES, Cerelli G, Oro A (1985). Primary structure and expression of a functional human glucocorticoid receptor cDNA.. Nature.

[46] Schuppan D, Krebs A, Bauer M, Hahn EG (2003). Hepatitis C and liver fibrosis.. Cell Death and Differentiation.

[47] Takahara Y, Takahashi M, Zhang Q–W, Wagatsuma H, Mori M (2008). Serial changes in expression of functionally clustered genes in progression of liver fibrosis in hepatitis C patients.. World J Gastroenterol.

[48] Gentile A, Trusolino L, Comoglio PM (2008). The Met tyrosine kinase receptor in development and cancer.. Cancer Metastasis Rev.

[49] Evangelista D, Colonna G, Miele M, Cutugno F, Castello G (2010). CDMS (Clinical Data Mining Software): a cytokinome data mining system for a predictive medicine of chronic inflammatory diseases.. PEDS.

