# Supplementary material for: Cytokinome Profile of Patients with Type 2 Diabetes and/or Chronic Hepatitis C Infection
Source: PLoS One. 2012 Jun 20;7(6):e39486. doi: 10.1371/journal.pone.0039486 (PMC3379982; doi:10.1371/journal.pone.0039486)
Supplement: Table S1 — List of molecules involved in the network reported in figure 4 . For each gene the table shows the symbol, Entrez Gene Name, Cellular Location and the type. (DOC) [file pone.0039486.s001.doc]

**Table S1. List of molecules involved in the network reported in figure 4.** For each gene the table shows the symbol, Entrez Gene Name, Cellular Location and the type.

| **Symbol** | **Entrez Gene Name** | **Location** | **Type(s)** |
| --- | --- | --- | --- |
| CASP1 | caspase 1, apoptosis-related cysteine peptidase | Cytoplasm | peptidase |
| CLU | clusterin | Extracellular Space | other |
| COPS5 | COP9 constitutive photomorphogenic homolog subunit 5 | Nucleus | transcription regulator |
| CXCL1 | chemokine (C-X-C motif) ligand 1 | Extracellular Space | cytokine |
| CXCL9 | chemokine (C-X-C motif) ligand 9 | Extracellular Space | cytokine |
| GHRL | ghrelin/obestatin prepropeptide | Extracellular Space | growth factor |
| IL18 | interleukin 18 | Extracellular Space | cytokine |
| IL18R1 | interleukin 18 receptor 1 | Plasma Membrane | transmembrane receptor |
| IL1A | interleukin 1, alpha | Extracellular Space | cytokine |
| IL2RA | interleukin 2 receptor, alpha | Plasma Membrane | transmembrane receptor |
| LEP | leptin | Extracellular Space | growth factor |
| MIF | macrophage migration inhibitory factor | Extracellular Space | cytokine |
| NR3C1 | nuclear receptor subfamily 3, group C, member 1 | Nucleus | ligand-dependent nuclear receptor |
| PIGR | polymeric immunoglobulin receptor | Plasma Membrane | transporter |
| PRDX1 | peroxiredoxin 1 | Cytoplasm | enzyme |
| RELA (NF-kB) | v-rel reticuloendotheliosis viral oncogene homolog A | Nucleus | transcription regulator |
| SERPINE1 | serpin peptidase inhibitor, clade E | Extracellular Space | other |
| STAT3 | signal transducer and activator of transcription 3 | Nucleus | transcription regulator |
| TP53 | tumor protein p53 | Nucleus | transcription regulator |
| USF1 | upstream transcription factor 1 | Nucleus | transcription regulator |
