# Supplementary material for: Cytokinome Profile of Patients with Type 2 Diabetes and/or Chronic Hepatitis C Infection
Source: PLoS One. 2012 Jun 20;7(6):e39486. doi: 10.1371/journal.pone.0039486 (PMC3379982; doi:10.1371/journal.pone.0039486)
Supplement: Table S2 — Ratio between the mean concentrations of significant molecules in LC versus CHC patients and in LCD vs CHD patients. We have evidenced in bold the ratio >1.5 (DOC) [file pone.0039486.s002.doc]

**Table S2.** **Ratio between the mean concentrations of significant molecules in LC versus CHC patients and in LCD vs CHD patients**. We have evidenced in bold the ratio >1.5
